# Incidence of and risk factors for tuberculosis among people with HIV on antiretroviral therapy in the United Kingdom

**DOI:** 10.1097/QAD.0000000000002599

**Published:** 2020-06-04

**Authors:** Clare L. van Halsema, Hajra Okhai, Teresa Hill, Caroline A. Sabin

**Affiliations:** a Department of Infectious Diseases and Tropical Medicine, North Manchester General Hospital, Manchester; b Liverpool School of Tropical Medicine, Liverpool; c Institute for Global Health; d National Institute for Health Research Health Protection Research Unit in Blood-Borne and Sexually Transmitted Infections, University College London, London, UK.

**Keywords:** antiretroviral therapy, CD4^+^ lymphocyte count, ethnic groups, HIV, tuberculosis

## Abstract

**Objective:**

The United Kingdom has a low tuberculosis incidence and earlier combination antiretroviral therapy (cART) is expected to have reduced incidence among people with HIV. Epidemiological patterns and risk factors for active tuberculosis were analysed over a 20-year period among people accessing HIV care at sites participating in the UK CHIC observational study.

**Design:**

Cohort analysis.

**Methods:**

Data were included for individuals over 15 years old attending for HIV care between 1996 and 2017 inclusive, with at least 3 months follow-up recorded. Incidence rates of new tuberculosis events were calculated and stratified by ethnicity (white/Black/other) as a proxy for tuberculosis exposure. Poisson regression models were used to determine the associations of calendar year, ethnicity and other potential risk factors after cART initiation.

**Results:**

Fifty-eight thousand seven hundred and seventy-six participants (26.3% women; 54.5% white, 32.0% Black, 13.5% other/unknown ethnicity; median (interquartile range) age 34 (29–42) years) were followed for 546 617 person-years. Seven hundred and four were treated for active tuberculosis [rate 1.3; 95% confidence interval (CI) 1.2–1.4/1000 person-years). Tuberculosis incidence decreased from 1.3 (1.2–1.5) to 0.6 (0.4–0.9)/1000 person-years from pre-2004 to 2011–2017. The decline among people of Black ethnicity was less steep than among those of white/other ethnicities, with incidence remaining high among Black participants in the latest period [2.1 (1.4–3.1)/1000 person-years]. Two hundred and eighty-three participants [191 (67%) Black African] had tuberculosis with viral load less than 50 copies/ml.

**Conclusion:**

Despite the known protective effect of cART against tuberculosis, a continuing disproportionately high incidence is seen among Black African people. Results support further interventions to prevent tuberculosis in this group.

## Introduction

Combination antiretroviral therapy (cART) is strongly protective against active tuberculosis in people with HIV across a range of settings [1–3]. The United Kingdom (UK) has a low incidence of tuberculosis and there is widespread access to cART, with the UNAIDS 90–90–90 target for HIV care being met for the first time in 2017 [4]. However, tuberculosis still occurs among individuals receiving cART and incidence has been shown to remain higher than that among people without HIV [5,6].

A previous analysis of the UK Collaborative HIV Cohort (UK CHIC) study, including data from 1996 to 2005, showed high incidence of tuberculosis among people with HIV of Black African ethnicity, despite the widespread availability of cART and improved CD4^+^ cell counts [7]. The risk of tuberculosis was the highest in those with low CD4^+^ cell counts and in those of Black African ethnicity. Since that time, tuberculosis incidence in the UK has decreased further [8]; people with HIV are living longer [9,10] and guidelines on the timing of cART have changed, with immediate rather than CD4^+^-guided start now recommended [11].

The UK CHIC cohort study provides an opportunity to examine the incidence of tuberculosis among people engaged in HIV care in the UK across a longer time period and to identify risk factors for the development of tuberculosis. We aimed to examine the incidence of tuberculosis and to identify risk factors for incident tuberculosis, particularly among those on cART.

## Methods

### Study participants

The UK CHIC study was initiated in 2001 and collates routine data on people with HIV, aged at least 16 years, who have attended one of 25 clinical centres providing HIV care in the UK at any time from 1996 onwards. Since 2004, the number of participating centres has increased from six to 25, with 10 in London, 14 in other parts of England and one in Edinburgh, Scotland. Participating centres are in large, urban areas. The study methods are described fully elsewhere [12]. In brief, centres collect data on demographic information, cART history, laboratory results, and AIDS diagnoses (including any tuberculosis events, classified as either pulmonary, extra-pulmonary or other/unknown), which are submitted on an annual basis to the co-ordinating centre. The analyses presented here include data collected up to 31 December 2017. Participants were eligible for analysis if they entered UK CHIC between 1996 and 2017, had a follow-up period of at least 3 months and at least 1 CD4^+^ T-cell count measurement after entry.

### Statistical analysis

Continuous variables were expressed as the median and interquartile range (IQR), and categorical variables as counts and percentages. Tuberculosis incidence was calculated based on the first tuberculosis episode recorded after entry to UK CHIC, regardless of any previous episodes that had been reported to occur prior to study entry. If tuberculosis was detected within 3 months of entry, we excluded follow-up time for that individual during the period of tuberculosis treatment (assumed to be 6 months after the tuberculosis episode date) to exclude tuberculosis disease that was most likely present at study entry and thus follow-up time for such individuals started 6 months after tuberculosis diagnosis.

To investigate factors independently associated with the occurrence of a tuberculosis event after starting cART, we used Poisson regression models. As this analysis was limited to those who initiated cART, follow-up started at cART initiation and ended at the earliest of date of tuberculosis episode, date of loss to follow-up or 31 December 2017. The date of the tuberculosis episode is defined as the date when the clinic reported tuberculosis as an AIDS event.

We examined associations with age, ethnicity (white; Black; other/unknown), a combined variable of sex and mode of HIV acquisition (sex between men; male heterosexuals; female heterosexuals; male other mode; female other mode), and clinical data after cART initiation (including CD4^+^ T-cell count, hepatitis B/C status and HIV viral load). Factors that were significantly associated with the risk of tuberculosis in univariable models were selected for inclusion in the multivariable model. In a subsequent analysis, we then further explored the effect of ethnicity on tuberculosis risk, using a more detailed breakdown of ethnicity: Black Caribbean, Black African, Black other, south Asian/other Asian, mixed/other and unknown.

### Ethical approval

UK CHIC was approved by a Multicentre Research Ethics Committee (MREC/00/7/47) and by local ethics committees.

## Results

### Description of cohort and tuberculosis events

Overall, 73 843 UK CHIC participants attended HIV clinics across the UK between 1996 and 2017. Of these, 933 individuals had at least one tuberculosis event after entry to UK CHIC, of which 57.9% were pulmonary tuberculosis. We excluded 15 067 study participants (229 tuberculosis events) because of insufficient follow-up time (*n* = 1490), missing CD4^+^ T-cell data (*n* = 2060) or both (*n* = 11 517), leaving 58 776 in the analyses with 704 tuberculosis events.

Study participants are described in Table 1. The median age at entry was 34 (interquartile range [IQR]: 29–42) years, but this increased from 33 years for those entering the study before 2004 to 37 years for those entering after 2011. The majority of study participants were of white ethnicity (54.5%), male (73.7%) and had a primary mode of HIV acquisition of sex between men (MSM, 52.3%). Participants were followed for a total of 546 617 person-years (PYRS) with a median follow-up time of 8.2 years [IQR: 3.6–13.8].

**Table 1:**
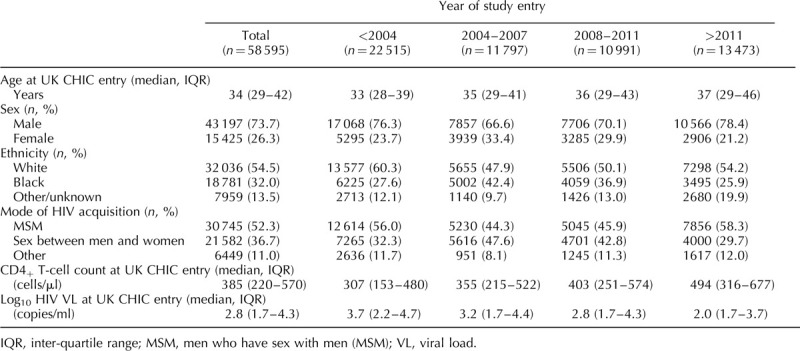
Characteristics of individuals included in analyses of tuberculosis incidence.

Median CD4^+^ T-cell count at study entry was 385 (IQR: 220–570) cells/μl, although this increased from 307 (IQR: 153–480) pre-2004 to 494 (IQR: 316–677) in the most recent time-period (post-2011). Similarly, the median HIV viral load at entry decreased over time from 3.7 (IQR: 2.2–4.7) log_10_ copies/ml pre-2004 to 2.0 (IQR: 1.7–3.7) log_10_ copies/ml post-2011.

In total, 704 individuals had at least one tuberculosis event over a median follow-up time of 3.4 (IQR: 1.4–6.6) years (incidence: 1.3/1000 PYRS, 95% confidence interval 1.2–1.4). Just over half of tuberculosis events were pulmonary events (56.5%), followed by extra pulmonary (38.8%) and other/unknown (4.7%) events. Most (58.1%, 409/704) tuberculosis events occurred after cART initiation with 69.2% (283/409) of these post-cART events occurring among individuals who had a suppressed (≤50 copies/ml) HIV viral load at the time of their tuberculosis episode. The median CD4^+^ T-cell count at the time of diagnosis was 260 (IQR 130–430) cells/μl. Though our analyses are restricted to the first tuberculosis event, 74 (10.5%) of these individuals had a subsequent tuberculosis episode recorded; individual characteristics at the time of these recurrent events were similar to those of the first tuberculosis event.

### Tuberculosis incidence over time

**Fig. 1:**
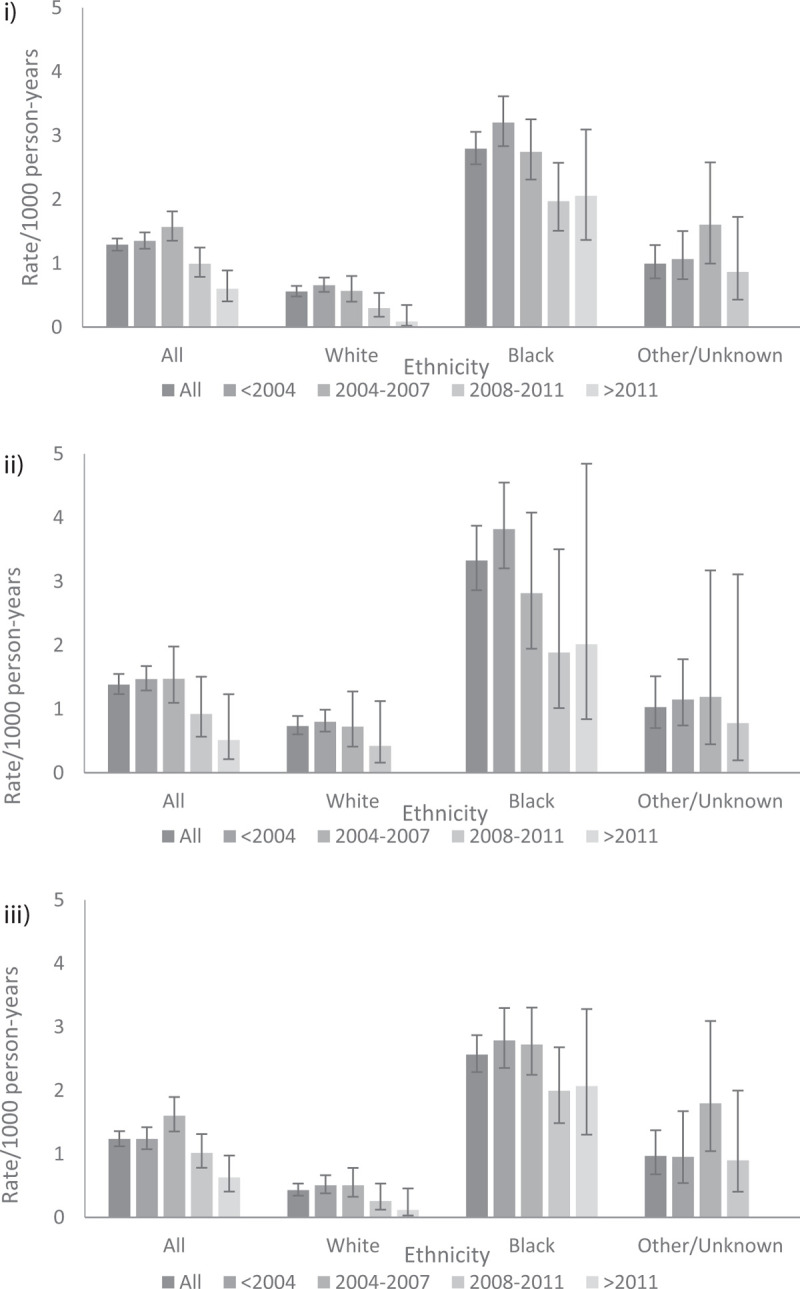
Tuberculosis incidence rate stratified by year of entry to UK CHIC and ethnicity in (i) all UK CHIC participants (tuberculosis events = 704), (ii) individuals not receiving cART (tuberculosis events = 295), and (iii) individuals receiving cART (tuberculosis events = 409).

The incidence of tuberculosis decreased over time (Fig. 1(i)), with an incidence in the most recent period (post-2011) of 0.6/1000 PYRS (95% CI 0.4–0.9) compared with 1.4/1000 PYRS (95% CI 1.2–1.5) in the pre-2004 period. The incidence of tuberculosis was the highest in those of Black ethnicity [2.8/1000 PYRS (95% CI 2.6–3.1)] compared with those of white (0.6/1000 PYRS [95% CI 0.5–0.7]) or other/unknown (1.0/1000 PYRS [95% CI 0.8–1.3]) ethnic groups. The decline over time was apparent in all ethnic groups, although was less marked in those of Black ethnicity than in the other groups. Patterns of decline were broadly similar regardless of whether individuals were untreated [295 events over 213 604 PYRS, Fig. 1(ii)] or had started cART [409 events over 331 893 PYRS, Fig. 1(iii)].

cART, combination antiretroviral treatment.

### Risk factors for tuberculosis among individuals taking combined antiretroviral therapy

In a univariable analysis of the 44 628 participants who started cART (Table 2), of 409 tuberculosis episodes after cART initiation, 21 (5.1%) occurred within 3 months; 29 (7.1%) 3–6 months after cART initiation; 51 (12.5%) 6–12 months after and 308 (75%) after 12 months on ART. Older participants were less likely to experience a tuberculosis event, as were those with a lower HIV viral load or higher CD4^+^ T-cell count over follow-up, those who initiated cART in more recent years and those who had been on cART for a longer period of time. MSM were the group with the lowest risk of a tuberculosis event when compared with all other sex/mode of HIV acquisition groups. Participants from the Black and other/unknown ethnic groups had a higher risk of tuberculosis compared with the white population. Most associations remained similar in the multivariable model (Table 3), with the exception of the association with age, which was attenuated and became nonsignificant after adjustment.

**Table 2:**
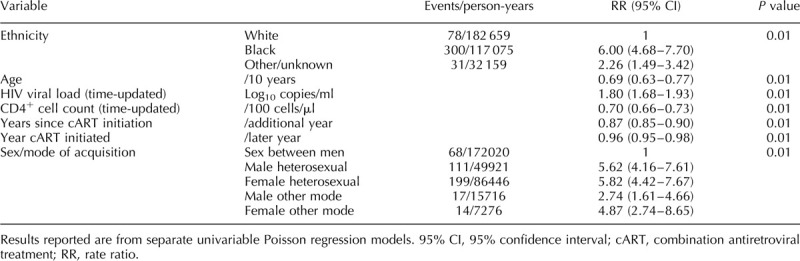
Analysis of risk factors for tuberculosis after starting combination antiretroviral treatment.

**Table 3:**
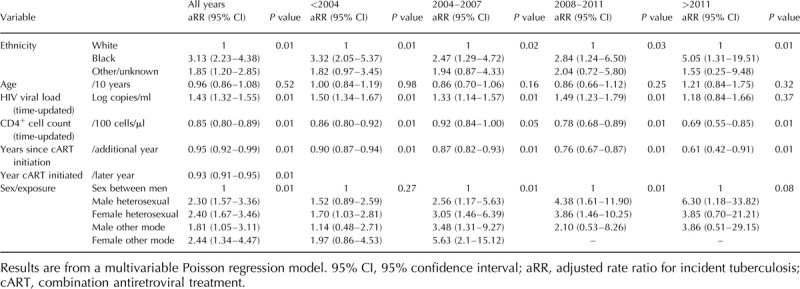
Analysis of risk factors for tuberculosis after starting combination antiretroviral treatment, overall and stratified by year of combination antiretroviral treatment initiation.

After stratifying the multivariable model by year of cART initiation (Table 3), the association with Black ethnic group appeared to strengthen over time, with an adjusted risk ratio in the post-2011 period (relative to the pre-2004 period) of 5.05 compared with values of 2.47–3.32 in the earlier periods. A test of interaction between Black ethnicity and calendar period confirmed that the rate in the Black ethnic group was significantly higher than would be expected based on the overall patterns for calendar period and ethnicity (*P* = 0.02 for interaction term). There is also a strong association between heterosexual route of HIV acquisition and persistently high tuberculosis incidence, with adjusted rate ratio for active tuberculosis of 2.3 (1.57–2.26) for male heterosexuals and 2.40 (1.67–3.46) for female heterosexuals compared with those reporting sex between men as the route of HIV acquisition. This is likely to be explained by residual confounding, with a large proportion of heterosexual adults (15 432/21 582 [71.5%]) being of Black African ethnicity. Of 18 781 people of Black African ethnicity, 15 432 (82.1%) report heterosexual acquisition.

**Fig. 2:**
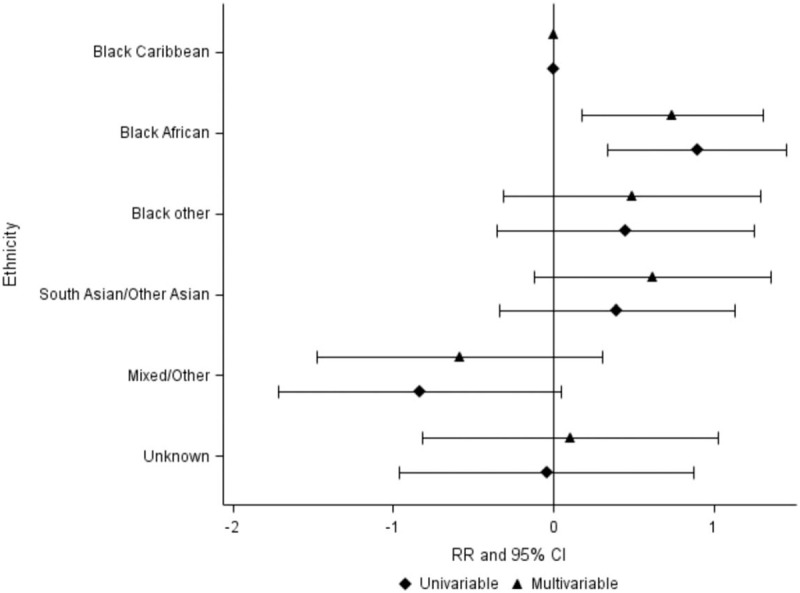
Unadjusted and adjusted^+^ relative risk of tuberculosis after starting combination antiretroviral treatment, ethnicity subgroup analysis.

As the white ethnic group had very low risk of tuberculosis, we performed a further analysis without this group to focus more on differences between minority ethnic groups (Fig. 2). In this analysis, including 331 tuberculosis episodes, occurring at median CD4^+^ cell count 299 cells/μl (IQR 170–489), ethnicity remained strongly associated with the risk of a tuberculosis event, with those of Black African origin having a higher risk of tuberculosis compared with those of Black Caribbean origin in both univariable and multivariable models [crude RR: 2.45 (1.40–4.27); adjusted RR: 2.10 (1.19–3.70)]. There was no evidence of a higher risk of tuberculosis among the other ethnic groups when compared with the Black Caribbean group.

Adjusted for age, HIV viral load, CD4^+^ cell count, years since cART initiation and year of cART initiation. 95% CI, 95% confidence interval; cART, combination antiretroviral treatment; RR, rate ratio.

## Discussion

In this UK cohort of people with HIV, tuberculosis still occurs among those receiving cART with incidence in the most recent time period (2011–2017) of 0.6/1000 PYRS compared with incidence in the general UK population at the time of around 0.13/1000 [8]. Incidence of tuberculosis among people with HIV has reduced with time; this is likely to be multifactorial, including association with overall reduction in tuberculosis incidence in the UK [8] and with the higher CD4^+^ cell count at entry to the study and earlier start of cART [11]. Public Health England data show that in 2017, 75% of those diagnosed with HIV with a CD4^+^ cell count greater than 350 cells/μl start cART within 91 days of diagnosis, compared with 30% in 2013 [4].

Ethnicity is a key risk factor for incident tuberculosis, with the Black African group continuing to have high incidence even after cART start and with this group not seeing the same fall in incidence evident in other ethnic groups. As expected, a lack of virological suppression and low CD4^+^ cell count are associated with incident tuberculosis but, despite this, most tuberculosis episodes occurred among individuals who were virologically suppressed on cART and most after 12 months on cART. The lack of data on birth or long-term residence in countries of higher tuberculosis incidence means that ethnicity used as a proxy for this may misclassify some and, therefore, the calculated measures of effect may be smaller than the true effect. It is known that, in countries of low tuberculosis incidence, a disproportionate number of incident tuberculosis cases occur among those born elsewhere, with slower decrease in incidence among individuals born outside those countries. This has been described in the UK as well as elsewhere in Europe and in the United States [8,13–15]. There are published strategies for targeted prevention, focused on identifying and treating latent tuberculosis infection among people moving from countries of high tuberculosis incidence to those of low incidence and people with HIV are a key group for whom this intervention should be provided [16,17].

In Table 3, an association is shown between heterosexual mode of HIV acquisition and tuberculosis risk, even after controlling for age, sex, ethnicity, CD4^+^ cell count and viral load. The epidemiology of HIV in the UK is such that the majority of people reporting sex between men as a route of acquisition are UK-born or of white ethnicity, or both, and the majority of people reporting heterosexual sex as mode of HIV acquisition are of Black African or other ethnicity or born in countries of high tuberculosis incidence. Route of HIV acquisition is not causally related to risk of active tuberculosis and the observed association is likely to be attributable to residual, unmeasured confounding.

Current guidance for diagnosis and treatment of latent tuberculosis among people with HIV in the UK is provided by the British HIV Association (BHIVA) and the National Institute for Health and Care Excellence (NICE) [18,19]. The 2011 BHIVA guidance [20] was in place during the most recent time period in this study, 2011–2017, and recommended testing for latent tuberculosis (and treating if present) according to epidemiological, immunological and cART criteria. The updated 2018 guideline [18] widens the recommendation to include all those from high and medium tuberculosis incidence countries and those from low incidence countries with additional risk factors or low CD4^+^ cell count. These recommendations are, however, poorly implemented [21,22] and missed opportunities for prevention of active tuberculosis are frequent [23], meaning that it is unlikely that the reduction in incidence seen in this analysis is attributable to treatment of latent tuberculosis.

This analysis builds on the previous UK CHIC paper [7], which included data up to 2005 and showed an increased risk of incident tuberculosis in people of Black African ethnicity compared with others, despite over 2 years of cART and a declining UK tuberculosis incidence at the time. Our findings are consistent with other analyses from countries with low tuberculosis incidence, in which greater tuberculosis risk is found among Black African, Asian and Hispanic participants with HIV than among white study participants with HIV [6,24]. This strongly suggests a greater need for prevention methods, including earlier HIV diagnosis and implementation of testing and treatment for latent tuberculosis infection according to existing guidelines. Treatment of latent tuberculosis along with cART for people with HIV is a well evidenced intervention, showing an effect in addition to cART on tuberculosis risk, including among individuals with higher CD4^+^ cell counts [25–27] albeit largely demonstrated in high tuberculosis-incidence settings. It is recommended by national and international guidelines [28] and in strategies for the elimination of tuberculosis in countries of low tuberculosis incidence [29].

A strength of this study is the large number of individuals included and follow-up time, the systematic data collection and good data on ethnicity and cART, with updated CD4^+^ cell counts. Limitations include the limited detail of clinical data on tuberculosis episodes, categorizing as pulmonary, extra-pulmonary or other/unknown, with unknown numbers microbiologically confirmed; the lack of data on place of birth or long-term residence and the use of ethnicity as a proxy for exposure to tuberculosis in a country of higher tuberculosis incidence. In addition, data are not collected on treatment of latent tuberculosis infection, although, as detailed above, coverage of this in the UK is thought to be low.

In conclusion, despite falling tuberculosis incidence in the UK, including among people with HIV, tuberculosis incidence remains disproportionately high among Black African people. This is despite the protective effect of cART and supports more widespread implementation of prevention strategies for those at risk.

## Acknowledgements

**The UK CHIC Study**

**Steering Committee:** Jonathan Ainsworth, Sris Allan, Jane Anderson, Ade Apoola, David Chadwick, Duncan Churchill, Valerie Delpech, David Dunn, Ian Fairley, Ashini Fox, Richard Gilson, Mark Gompels, Phillip Hay, Rajesh Hembrom, Teresa Hill, Margaret Johnson, Sophie Jose, Stephen Kegg, Clifford Leen, Dushyant Mital, Mark Nelson, Hajra Okhai, Chloe Orkin, Adrian Palfreeman, Andrew Phillips, Deenan Pillay, Ashley Price, Frank Post, Jillian Pritchard, Caroline Sabin, Achim Schwenk, Anjum Tariq, Roy Trevelion, Andy Ustianowski, John Walsh.

**Central Co-ordination:** University College London (David Dunn, Teresa Hill, Hajra Okhai, Andrew Phillips, Caroline Sabin); Medical Research Council Clinical Trials Unit at UCL (MRC CTU at UCL), London (Nadine van Looy, Keith Fairbrother).

**Participating Centres:** Barts Health NHS Trust, London (Chloe Orkin, Janet Lynch, James Hand); Brighton and Sussex University Hospitals NHS Trust (Duncan Churchill, Stuart Tilbury, Elaney Youssef, Duncan Churchill); Chelsea and Westminster Hospital NHS Foundation Trust, London (Mark Nelson, Richard Daly, David Asboe, Sundhiya Mandalia); Homerton University Hospital NHS Trust, London (Jane Anderson, Sajid Munshi); King's College Hospital NHS Foundation Trust, London (Frank Post, Ade Adefisan, Chris Taylor, Zachary Gleisner, Fowzia Ibrahim, Lucy Campbell); Middlesbrough, South Tees Hospitals NHS Foundation Trust, (David Chadwick, Kirsty Baillie); Mortimer Market Centre, University College London (Richard Gilson, Ian Williams); North Middlesex University Hospital NHS Trust, London (Jonathan Ainsworth, Achim Schwenk, Sheila Miller, Chris Wood); Royal Free NHS Foundation Trust/University College London (Margaret Johnson, Mike Youle, Fiona Lampe, Colette Smith, Rob Tsintas, Clinton Chaloner, Caroline Sabin, Andrew Phillips, Teresa Hill, Hajra Okhai); Imperial College Healthcare NHS Trust, London (John Walsh, Nicky Mackie, Alan Winston, Jonathan Weber, Farhan Ramzan, Mark Carder); The Lothian University Hospitals NHS Trust, Edinburgh (Clifford Leen, Andrew Kerr, David Wilks, Sheila Morris); North Bristol NHS Trust (Mark Gompels, Sue Allan); Leicester, University Hospitals of Leicester NHS Trust (Adrian Palfreeman, Adam Lewszuk); Woolwich, Lewisham and Greenwich NHS Trust (Stephen Kegg, Victoria Ogunbiyi, Sue Mitchell), St. George's Healthcare NHS Trust (Phillip Hay, Christopher Hunt, Olanike Okolo, Benjamin Watts); York Teaching Hospital NHS Foundation Trust (Ian Fairley, Sarah Russell-Sharpe, Olatunde Fagbayimu); Coventry, University Hospitals Coventry and Warwickshire NHS Trust (Sris Allan, Debra Brain); Wolverhampton, The Royal Wolverhampton Hospitals NHS Trust (Anjum Tariq, Liz Radford, Sarah Milgate); Chertsey, Ashford and St. Peter's Hospitals NHS Foundation Trust (Jillian Pritchard, Shirley Cumming, Claire Atkinson); Milton Keynes Hospital NHS Foundation Trust (Dushyant Mital, Annie Rose, Jeanette Smith); The Pennine Acute Hospitals NHS Trust (Andy Ustianowski, Cynthia Murphy, Ilise Gunder); Nottingham University Hospitals NHS Trust (Ashini Fox, Howard Gees, Gemma Squires, Laura Anderson), Kent Community Health NHS Foundation Trust (Rajesh Hembrom, Serena Mansfield, Lee Tomlinson, Christine LeHegerat, Roberta Box, Tom Hatton, Doreen Herbert), The Newcastle upon Tyne Hospitals NHS Foundation Trust (Ashley Price, Ian McVittie, Victoria Murtha, Laura Shewan); Derby Teaching Hospitals NHS Foundation Trust (Ade Apoola, Zak Connan, Luke Gregory, Kathleen Holding, Victoria Chester, Trusha Mistry, Catherine Gatford); Public Health England, London (Valerie Delpech); i-Base (Roy Trevelion).

**The NIHR HPRU in Blood Borne and Sexually Transmitted Infections Steering Committee:** Caroline Sabin (Director), John Saunders (PHE Lead), Catherine Mercer, Gwenda Hughes, Sema Mandal, Greta Rait, Samreen Ijaz, Tim Rhodes, Kholoud Porter, and William Rosenberg.

Funding: The UK CHIC Study is funded by the Medical Research Council, UK (grant numbers G0000199, G0600337, G0900274 and M004236). The study is also supported by an NIHR Senior Investigator Award to C.A.S. and through the National Institute for Health Research Health Protection Research Unit (NIHR HPRU) in Blood Borne and Sexually Transmitted Infections at University College London in partnership with Public Health England (PHE), in collaboration with London School of Hygiene & Tropical Medicine (LSHTM). The views expressed are those of the authors and not necessarily those of the NIHR, the Department of Health and Social Care or Public Health England.

### Conflicts of interest

C.A.S. has received financial support for the membership of Data Safety and Monitoring Boards, Advisory Boards and for preparation of educational materials from Gilead Sciences and ViiV Healthcare. C.L.v.H. has received financial support for membership of Advisory Boards, conference attendance, speaker fees and educational grants from ViiV healthcare, MSD Ltd, Oxford Immunotec and Gilead Sciences.
